# Aging, Metabolism, Synaptic Activity, and Aβ in Alzheimer's Disease

**DOI:** 10.3389/fnagi.2019.00185

**Published:** 2019-07-23

**Authors:** Gunnar K. Gouras

**Affiliations:** Experimental Dementia Research Unit, Department of Experimental Medical Science, Lund University, Lund, Sweden

**Keywords:** Amyloid-β protein, synapse, tau protein, endosome, neuropathology, lipids, Alzheimer's disease

Aging is the leading risk factor for the development of Alzheimer's disease (AD). However, the molecular and cellular mechanisms critical for how aging impacts AD remain unclear. The brain uses a large proportion of energy, with synaptic transmission being its main source of energy use (Harris et al., 2012). Synapses are early sites of damage in AD and reduced brain metabolic activity is a well-known outcome of the disease. Synaptic activity has been linked to AD by observations that brain areas with high metabolic activity within the default mode network are particularly prone to the development of AD. Synaptic activation in turn impacts levels of central peptides/proteins linked to AD, including β-amyloid (Aβ) and tau, the main components of the two neuropathological hallmarks of the disease, the amyloid plaques and neurofibrillary tangles, respectively. Synaptic activity modulates the biology of these aggregation-prone proteins and impacts their brain pathologies. A better understanding of the interplay between brain metabolic activity and protein aggregation at synapses will be important to better understand neurodegenerative diseases of aging.

Aging impacts all organs and biological pathways, and is a risk factor for numerous systemic diseases from atherosclerosis to cancers. Aging promotes a large class of diseases of the nervous system that are particularly feared, the neurodegenerative diseases of aging, including the most common and widely known Alzheimer's disease (AD) and Parkinson's disease (PD). Common themes shared among diverse age-related neurodegenerative diseases are aberrant protein aggregations, early synapse dysfunction and damage, and selective neuronal vulnerabilities. Although vascular disease risk factors, such as obesity and diabetes, increase the risk for dementia (Kivipelto et al., 2005), there are less clear links between vascular risk factors and the more characteristic biomarker and pathological alterations that reflect AD. Thus, while vascular risk factors influence cognitive decline with age, their direct influences on AD pathophysiology remain less well-established.

Energy metabolism in the aging brain is affected by numerous factors. While the mechanisms whereby altered energy metabolism with aging influence early protein accumulations/aggregations with neurodegenerative diseases are of major interest, protein aggregation in turn can impact brain metabolism at an early stage of disease. Among the earliest changes described in AD brains are enlargement of endosomes (Cataldo et al., 2000), neuronal Aβ accumulation (Gouras et al., 2000), and synaptic alterations (DeKosky and Scheff, 1990; Masliah et al., 1994). How these biological alterations in Aβ, endosomes and synapses relate to each other and how altered energy metabolism with aging might promote these alterations requires further research.

## Aβ42 as an Early Mediator of Dysfunction in AD

Multiple lines of evidence have pointed to an important role for Aβ in the development of AD. Next to mutations in the amyloid precursor protein (APP) linked with early onset autosomal dominant familial forms of AD, reduction of levels of the disease-linked Aβ42 form of the peptide in the cerebrospinal fluid (CSF) is the earliest known biomarker heralding future AD many years before the first symptoms arise in all known forms of AD (Bateman et al., 2012; Buchhave et al., 2012). This drop in CSF Aβ42 is then followed by the emergence of amyloid plaques by PET ligand amyloid imaging (Palmqvist et al., 2017) and typically several years later by a gradual increase in levels of total and phosphorylated tau in the CSF, which appears closer to the time of initial cognitive symptoms. However, the wet and imaging biomarker data contrast with the emergence of the hallmark pathologies in AD brains. Here tau tangles in the brain hemispheres initially form in the “transentorhinal” cortex, while amyloid plaques are seen later, appearing initially in frontal cortex (Braak and Braak, 1991; Thal et al., 2002).

A newer but controversial insight into early AD pathology, which can also help explain the different CSF biomarker and brain pathology trajectories, as well as the anatomical disconnect between Aβ and tau pathologies, was the finding of early accumulation of Aβ42 within AD vulnerable neurons (Gouras et al., 2000). Although this Aβ42 accumulation is modest, and thus was overlooked, numerous papers have since replicated these findings in human AD and Down syndrome brains as well as in numerous transgenic rodent models of AD (reviewed in Gouras et al., 2010; see also Kobro-Flatmoen et al., 2016; Welikovitch et al., 2018). In our further modified version of the amyloid cascade hypothesis, this initially modest intracellular Aβ42 noticeable in cell bodies of AD vulnerable neurons early on leads then to more prominent synaptic/neuritic Aβ aggregation, endosomal and synapse alterations, and alterations in tau (Takahashi et al., 2002, 2004, 2010; Willén et al., 2017). Thus, AD vulnerable neurons go from intracellular Aβ42 increases evident at cell soma, progress to pathological Aβ aggregation at terminals, somatodendritic tau aggregation and hyperphosphorylation, and eventually cell death of vulnerable neurons, including entorhinal cortex (ERC) layer 2 stellate neurons, basal forebrain cholinergic neurons and noradrenergic projection neurons in the locus ceruleus, among others. The anatomical disconnect between amyloid and tau pathologies arises, because Aβ42 aggregation and formation of plaques occurs particularly in the terminal fields of such AD vulnerable neurons, both in distal axons and dendrites, Aggregating Aβ42 eventually breaks through the cell membrane of terminals to become extracellular (Willén et al., 2017), whereupon it can act as a nidus for plaque formation recruiting also secreted extracellular Aβ. Thus, for example, ERC layer 2 neuron axon terminals in the hippocampal dentate gyrus as they degenerate from intracellular Aβ42 aggregation give rise to “extracellular” plaques, while their corresponding cell soma develop tangles and then die.

## Synaptic Activity and Synapse Damage in AD

The role of synaptic activity in synapse damage and plaque formation is complex. Alzheimer brain dysfunction initiates particularly in areas known to be more chronically active—the so-called default network. Both excessive activity from stress or seizures can worsen brain damage in transgenic mouse models of brain β-amyloidosis (Mohajeri et al., 2002), while excessive sleep or reduced brain activity also damage synapses in such AD models but not wild-type mice (Tampellini et al., 2010). Thus, brain activity at either too high or low levels damages synapses in the setting of excessive Aβ. These studies further underscore that plaques are not the fundamental cause of synapse damage. It was long known that amyloid plaques are not a good correlate of cognitive decline in human AD, and numerous experimental studies have shown discordance between plaques and cognitive decline. For example, the initial active vaccine clinical trial against Aβ pointed to engagement of the antibodies by plaque reduction via microglia degradation without however any corresponding cognitive improvement (Holmes et al., 2008). Moreover, an inducible mouse model of amyloidosis showed rapid improvement in behavior despite persistence of plaques when the AD mutant APP transgene was turned off (Melnikova et al., 2013). In the setting of reduced brain activity via either unilateral whisker removal or chronic benzodiazepine treatment, there was loss of synaptic terminals despite reduction in amyloid plaques (Tampellini et al., 2010). This supports an emerging view that intracellular Aβ42 aggregates in dystrophic neurites are the synaptotoxic Aβ entities that form the nidus of eventual plaques, which however lead to the large amyloid plaques from the contribution of secreted Aβ. Disruption of circuitry in the brain leads to inhibitory-excitatory imbalances, resulting in hypo- and hyper-excitable neurons. Transgenic AD mice overproducing Aβ show hyper-activation as detected by calcium oscillations even prior to amyloid plaques, while tau transgenic mice show hypo-excitation even prior to tangles (Busche et al., 2019), underscoring the pathogenic roles of more soluble forms of both Aβ and tau prior to their neuropathological AD hallmark lesions traditionally viewed as the toxic aggregates.

## How Does Aging Promote Proteinopathy and Can Therapy Intervene

A central unanswered question in AD is how the most important AD risk factor, aging influences the characteristic proteinopathies in AD. The mitochondrial free radical theory of aging has been extensively studied with regards to how aging impacts AD and other neurodegenerative diseases. However, like anti-amyloid approaches, knowledge about mitochondria and free radicals has so far not led to therapeutic success for AD. Overall, more effort has gone into how Aβ impacts mitochondria and oxidative stress rather than how these can influence Aβ aggregation with age. However, induction of oxidative stress by crossing of heterozygous MnSOD^+/−^ mice with AD transgenic mice showed enhanced amyloid pathology (Li et al., 2004), supporting that oxidative stress can induce Aβ pathology rather than only the other way around. Given that other age-related diseases such as atherosclerosis have also shown more therapeutic success so far by targeting aberrant accumulation, albeit of lipids, rather than targeting mitochondria or free radicals, it likely also makes sense to focus first on modulating proteinopathies in the quest for therapy of neurodegenerative diseases. Nevertheless, in the future, as knowledge about altered metabolism with aging advances, targeting these more upstream aging mechanisms, such as via protection against free radicals and/or by mitochondrial rejuvenation, are compelling therapeutic directions.

Experimental therapeutic studies have also shown that central signaling pathways important for aging can be targeted for improvement in AD transgenic models, including modulation of mTOR (Caccamo et al., 2010; Ma et al., 2010; Spilman et al., 2010) and AMPK (Wang et al., 2019), among others. However, such central signaling pathways are also implicated in synaptic function and therefore need to be approached with caution. These signaling pathways link to another pathological hallmark of neurodegenerative diseases, the abnormal accumulation of autophagic vesicles, particularly evident in dystrophic neurites in AD brains. Impaired degradation likely significantly contributes to the proteinopathies that characterize neurodegenerative diseases, and therapies aimed at promoting autophagy-lysosome degradation are another important direction to pursue. Metabolism is intricately linked with this degradation system; for example, experimental stimulation of TFEB can promote degradation of protein aggregates. Evidence also points to Aβ-dependent impairment of the ubiquitin proteasome system and endosome-lysosome-autophagy pathway, degradation pathways whose biology are linked (Almeida et al., 2006; Willén et al., 2017).

## Lipids and Age-Related Neurodegenerative Diseases

Although research on lipids in AD has been minimal compared to that on protein aggregates, apolipoprotein E (apoE) 4 is the major genetic risk factor for AD, and thereby strongly implicates lipids in the disease (Huynh et al., 2017). Lipids are known to accumulate in brain with aging and AD, however, further mechanistic insights will be necessary for therapeutic targeting of lipids for AD. Lipids might also directly intersect with protein aggregates of AD in that lipids are taken up by neurons via apoE receptors via endocytosis and could therefore intersect with e.g., accumulating Aβ within endosomes. Endosomes were shown to be sites of initial Aβ accumulation (Takahashi et al., 2002) and numerous AD genes point to vesicular trafficking and endocytic pathway involvement in AD (Small et al., 2017; Guimas Almeida et al., 2018). Particularly trafficking at distal neurites may be vulnerable sites for AD, where synaptic activity, protein aggregation, energy metabolism/oxidative stress, lipids and endosome-lysosome-autophagy system intersect.

## Conclusion

Research on age-related neurodegenerative diseases is advancing but so far has not been able to translate this progress into effective therapy. In contrast, many other systemic diseases induced by aging have had remarkable success in developing new therapies. A major difference between these diseases is that neurodegenerative diseases occur in the least understood organ of our body, the brain, and how synapses function and interact within complex anatomical circuits remains a final frontier for neuroscience research. Aging plays a central role in age-related neurodegenerative diseases, where synapses are now seen as particularly vulnerable sites of attack. Further understanding of the precise steps leading from age-related metabolic impairment and mitochondria dysfunction to early protein aggregation at synapses will be of major importance to unravel the mysteries of age-related neurodegeneration. Future therapeutic research will also need to delve more into defective lipid biology with aging in different types of brain cells, including next to neurons also astrocytes and microglia, that appear to intersect with protein aggregation to make synapses so vulnerable in AD.

## Author Contributions

The author confirms being the sole contributor of this work and has approved it for publication.

### Conflict of Interest Statement

The author declares that the research was conducted in the absence of any commercial or financial relationships that could be construed as a potential conflict of interest.
